# Corrosion Behavior of Surface-Treated Metallic Implant Materials

**DOI:** 10.3390/ma13092011

**Published:** 2020-04-25

**Authors:** Therese Bormann, Phuong Thao Mai, Jens Gibmeier, Robert Sonntag, Ulrike Müller, J. Philippe Kretzer

**Affiliations:** 1 Laboratory of Biomechanics and Implant Research, Clinic for Orthopedics and Trauma Surgery, Heidelberg University Hospital, 69118 Heidelberg, Germany; 2 Institute for Applied Materials, Karlsruhe Institute of Technology, 76131 Karlsruhe, Germany

**Keywords:** implant, biomaterial, corrosion, residual stress, total hip replacement, taper connection, anodic polarization, surface treatment

## Abstract

Corrosion of taper connections in total hip arthroplasty remains of concern, as particles and ions generated by corrosive processes can cause clinical problems such as periprosthetic osteolysis or adverse reaction to metallic debris. Mechanical surface treatments that introduce compressive residual stresses (RSs) in metallic materials can lead to a better performance in terms of fretting and fatigue and may lower the susceptibility to corrosion. The study investigates the impact of mechanical surface treatments on the corrosion behavior of metallic biomaterials. Compressive RSs were introduced by deep rolling and microblasting in Ti6Al4V and CoCrMo samples. Polished samples served as reference. Corrosion behavior was characterized by repeated anodic polarization. Residual stresses of up to about −900 MPa were introduced by deep rolling with a reach in depth of approximately 500 µm. Microblasting led to compressive RSs up to approximately −800 and −600 MPa for Ti6Al4V and CoCrMo, respectively, in the immediate vicinity of the surface. For Ti6Al4V, microblasting resulted in decreased corrosion resistance with lower breakdown potentials and/or increased passive current densities in comparison to the polished and deep-rolled samples. The corrosion behavior of CoCrMo on the other hand was not affected by the mechanical surface treatments.

## 1. Introduction

Corrosion of metallic parts of artificial joints remains of concern as ions and particles that are generated by the corrosive processes can lead to clinical problems [1,2]. Alloys based on titanium and cobalt-chromium or stainless steels are usually applied as implant materials. The alloys themselves are highly corrosion resistant as they are passive metals (i.e., a protective oxide layer insulates the metal from the environment preventing corrosion). However, if the passive layer is continuously damaged, corrosion can—and does—occur.

In endoprosthetic implants, modularity enables intraoperative flexibility, which is especially beneficial in case of revision operations. In total hip arthroplasty (THA), the most prominent modular example is the taper connection between hip stem and femoral head. The material combination Ti6Al4V–CoCrMo is commonly used in these taper connections, because hip stems are often manufactured from Ti6Al4V due to its relatively low elastic modulus and the good osseointegrative properties, while metallic femoral heads are usually made of CoCrMo because of its high abrasion resistance. Corrosive processes at this interface are often referred to as *mechanically assisted crevice corrosion* (MACC) [3]. This means that micromotion caused by cyclic implant loading leads to passive layer damage, which, in turn, promotes fretting wear as well as galvanic and crevice corrosion. In extreme cases, almost the complete trunnion of the taper connection was found to be worn off (trunnionosis) [4,5,6]. However, even in less dramatic cases, a considerable amount of metal loss can be generated at the head–neck interface, which can cause clinical problems, such as periprosthetic osteolysis and adverse reaction to metallic debris [7]. Furthermore, elevated blood metal ion levels can be provoked by corroding taper connections [7]. Using ceramic heads instead of heads from CoCrMo is a possibility to reduce corrosion at this particular interface. However, also for ceramic heads on metallic bone stems, taper corrosion has been reported, albeit to a lower extent [8]. Independent of head material, state-of-the-art to prevent taper corrosion is a proper head impaction during surgery. It has been shown that an impact power of at least 4 kN should be applied to ensure good interlocking of head and stem and reduce micromotion as far as possible [9,10,11,12]. 

Another approach in increasing fatigue and corrosion resistance is to alter the surface properties of the metals. The induction of compressive residual stresses in the surface and subsurface region reduces the susceptibility to crack initiation, for example by nanocrystal formation and strain hardening, which, in turn, enhances the resistance towards fatigue and fretting fatigue [13,14,15]. For Ti6Al4V, a superior fatigue and fretting fatigue performance after shot peening processes has been shown, for example, by Sonntag et al., Altenberger et al. and Liu et al. [14,16,17]. Aside from the mechanical behavior, corrosion characteristics of metals are influenced by residual stresses. It is well known that tensile RSs increase the susceptibility of stress corrosion cracking. Compressive RSs, on the other hand, can stop crack propagation and decrease the susceptibility of pitting corrosion [18,19,20]. The beneficial effects of compressive RSs on the corrosion resistance were shown for example for AA2024-T3—an aluminum alloy with poor corrosion properties—and a magnesium alloy for biomedical applications [19,20]. Lee at al. investigated the effect of shot peening on Ti6Al4V, which is a well-known technique to induce compressive RSs, and found increased high-cycle fatigue resistance even under seawater environment [21]. Both by shot peening and deep rolling, compressive RSs can be induced for depth ranges of some 100 µm depending on the process parameters used. However, techniques like deep rolling or laser shock peening have likewise been applied to induce compressive RSs to depths of up to a millimeter and even more depending on the intensity of the process [16]. 

This study aims at investigating the corrosion properties of Ti6Al4V and CoCrMo after inducing compressive RSs by the techniques of deep rolling and microblasting. The corrosion behavior of the mechanically surface-treated materials is investigated by means of anodic polarization. The two investigated alloys are commonly used as pairing in head–neck taper connections in total hip arthroplasty. As corrosion at this interface is complex, for separation of the individual influencing factors in this first approach, only the material–electrolyte interaction was investigated. We are aiming to assess the potential of mechanical surface treatments with regard to improved corrosion resistance of modular taper junctions in THA. 

## 2. Materials and Methods 

Surface treatments and electrochemical corrosion experiments were performed on discs made of Ti6Al4V ELI (Ti) as defined in DIN ISO 5832-3 and the low carbon CoCrMo-alloy (CoCr) defined in DIN ISO 5832-12, respectively. Ti6Al4V was received as rod material with a diameter of 15 mm. To provide a defined microstructure and to eliminate all residual stresses, the rod material was heat treated at 900 °C for 10 min under vacuum followed by oil quenching. Finally, the material was annealed at 500 °C for 4 h followed by a slow furnace cooling. CoCrMo was received as rod material with a diameter of 28 mm. This material was used in the as-received state. For surface treatments and electrochemical tests, discs with a diameter of 15 mm (Ti) and 28 mm (CoCr) and a thickness of 3 mm were manufactured. 

### 2.1. Surface Treatments 

The corrosion behavior of the two materials was investigated for the three different surface conditions of (i) metallographical polishing (PO), (ii) deep rolling (DR) and (iii) microblasting (MB). 

A metallographic preparation (i.e., grinding, fine grinding and polishing by diamond and/or oxide suspension) removes the residuals from previous processing. Therefore, the metallographically-prepared samples served as reference. The mechanical surface treatments of deep rolling and microblasting on the other hand induced significant compressive residual stresses. Microblasting is a shot peening process with grit sizes in the micrometer range and a commonly used technique for surface treatments of metallic implant materials [22,23]. 

All samples were at first on one face ground and fine ground using SiC-paper from a grit of P320 to finally P2500. For the polished samples, the final surface treatment comprised diamond polishing applying diamond suspension down to a grain size of 3 µm.

Deep rolling and microblasting were carried out according to the process parameters listed in Table 1. 

**Figure 1:**
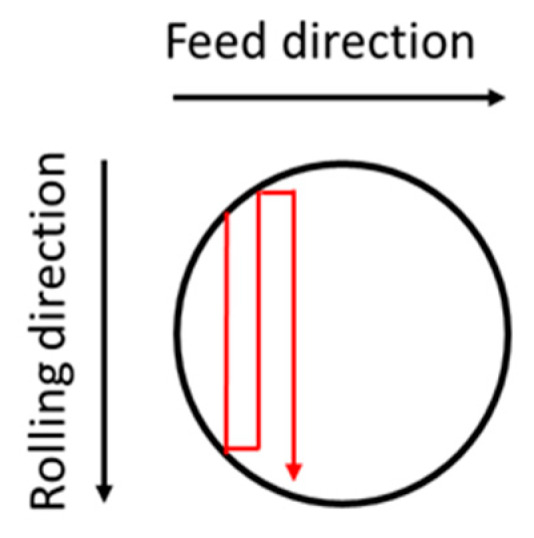
Scheme of the meandering pattern used for deep rolling and microblasting with indication of the feed and the rolling direction.

For the deep rolling, the rolling tool is directed using a meandering pattern, as schematically shown in Figure 1. Here, the compressive residual stresses at the material’s surface are generated through continuous plastic deformation induced through the rolling contact (Hertzian pressure) between the tool and the workpiece material. The overlapping of adjacent rolling tracks induces characteristic non-axisymmetric residual stress distributions in lateral direction, i.e., maximum compressive residual stresses occur in feed direction, while in rolling direction, typically, much lower compressive residual stresses develop.

Microblasting is carried out by moving the blast nozzle also following a meandering pattern. Each blasting grain induces a residual imprint due to plastic deformation of the interacting material. Due to the high coverage the resulting lateral compressive residual stress distribution in the near-surface region is generally independent of direction (i.e., an axisymmetric residual stress state is generated). 

The results of the surface treatments were assessed by X-ray residual stress analysis.

Scanning electron microscopy (LEO EVO 50, Zeiss, Oberkochen, Germany) in secondary electron contrast mode was applied in order to visualize the sample surfaces after the final surface treatment. 

### 2.2. X-ray Residual Stress Analysis

Residual stresses in the two principal directions were determined using a custom-made 3-axis X-ray diffractometer in ψ-configuration according to the well-known sin²ψ-technique [24]. X-ray diffraction analysis was performed with Ni-filtered Cu Kα radiation for the {213}-lattice planes in the 2Θ-range 136° ≤ 2Θ ≤ 147.4°. Fifteen sample tilts were considered in the range −60° ≤ ψ ≤ 60°. As primary aperture, a ø 1 mm pin hole collimator was applied. On the secondary side, a 4 mm symmetrizing slit was used in front of the scintillation counter. Data post-processing was carried out using a Pearson VII fit after background subtraction. The diffraction elastic constants (DEC) E_{213}_ = 113 GPa and ν_{213}_ = 0.32 for Ti6Al4V and E_{220}_ = 227 GPa and ν_{220}_ = 0.3 for the CoCr samples were applied. Depth distributions of residual stresses were determined by means of electrochemical sublayer removal and reapplication of the sin²ψ-measurement at the newly created surface. 

### 2.3. Electrochemical Testing

Electrochemical tests were accomplished using a potentiostat (Wenking MLab 200, Bank Elektronik – Intelligent Controls, Pohlheim, Germany) in combination with an electrochemical cell. The standard three-electrode system was applied for electrochemical testing with the Ti or CoCr discs serving as working electrode. Discs were covered by a silicone sealing mask which exposed the metal to the electrolyte through an orifice of 11.3 mm diameter, resulting in a working electrode area of 1 cm². The KCl saturated Ag/AgCl reference electrode (Sensortechnik Meinsberg, Waldheim, Germany) was placed in a Luggin capillary. The distance between capillary tip and working electrode was set to 1 mm. A platinized titanium bolt of 6 mm diameter served as counter electrode. Anodic polarization curves were recorded between −800 and 2000 mV for Ti and between −800 and 1000 mV for CoCr samples. Anodic polarization was repeated four times for each sample. The scan rate was set to 1 mV/s as proposed in the ASTM standard F2129-15 [25]. Open-circuit potential (OCP) measurements were conducted before, in between and after the polarization scans for one hour in each case. Dulbecco’s phosphate buffered saline (PBS) (Biochrom, Berlin, Germany) with a pH of 7.4 was used as electrolyte for electrochemical tests of the three generated surface conditions for Ti and CoCr samples. In addition, a second electrolyte containing 10 g/l FeCl_3_ in Ringer’s solution (FeCl_3_) (B. Braun AG, Melsungen, Germany) was used for electrochemical tests of polished, deep-rolled and microblasted Ti discs. The FeCl_3_-containing electrolyte has a low pH of 1.8 and a higher concentration of Cl^−^ ions and was used as corrosion accelerating environment [26,27]. Electrochemical testing was done at 37 °C and by using aerated electrolytes. Before electrochemical testing, all samples were ultrasonically cleaned in ethanol for 10 min. The current density of the passive plateau (I_p_) was determined from the anodic polarization curves as qualitative measurement of material degradation processes. The breakdown potential (E_b_), which characterizes the onset of pitting corrosion, was determined from the intersection between passive current density and a linear fit of the polarization curve in the transpassive regime (i.e., the part of the curve leaving the passive plateau abruptly towards higher current density values). All curves were median filtered prior to the determination of I_p_ and E_b_. 

### 2.4. Surface Roughness Measurements

Surface roughness measurements were carried out after electrochemical testing using a perthometer (M2, Mahr, Göttingen, Germany). For each sample, four measurements were done in the area exposed to the electrolyte as well as in sample regions that were not exposed to the electrolyte during electrochemical testing but were covered by a silicone sealing sheet. The direction of the surface roughness profiles was changed about 90° for each measurement. The first roughness profile was placed arbitrarily on the sample surface. We consider the unexposed surface region to reflect the initial surface roughness of the samples before electrochemical testing. The length of the measuring line was 1.75 mm. 

All results are given as mean ± standard deviation from three samples (n = 3). 

## 3. Results

### 3.1. Mechanical Surface Treatment

**Figure 2:**
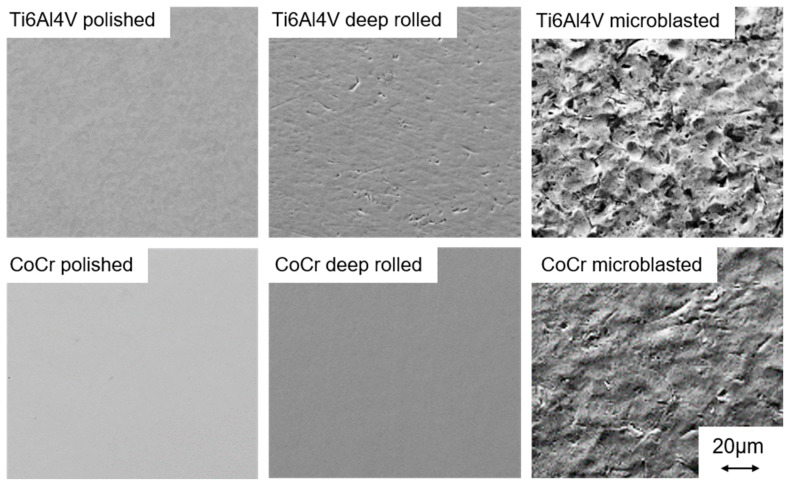
Scanning electron microscope images of the polished, deep-rolled and microblasted Ti6Al4V and CoCrMo (CoCr) samples.

The scanning electron microscope (SEM) images presented in Figure 2 emphasize the different characteristics of the mechanical surface treatments. Compared to the polishing and deep rolling process, microblasting results in a more rugged surface due to the stochastic impact of individual grits.

**Figure 3:**
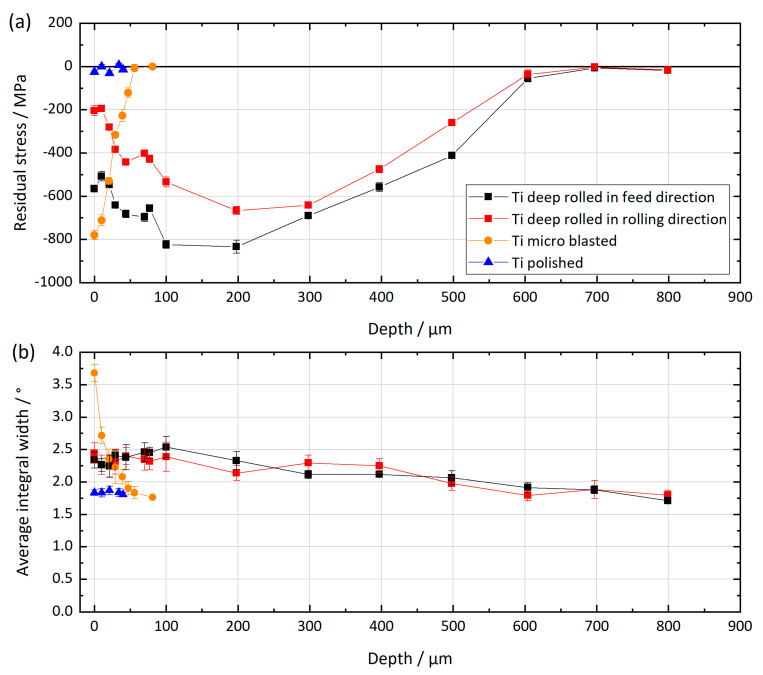
Residual stress depth distributions (**a**) and depth distributions of the average integral widths of the diffraction lines (**b**) for the three differently mechanically surface-treated Ti6Al4V (Ti) samples.

The residual stress depth distributions for the Ti6Al4V samples are presented in Figure 3 together with the depth distributions of the mean integral widths of the recorded diffraction lines. In comparative studies, using the same measuring parameters, the change in the integral widths values corresponds to the degree of cold working (i.e., higher values indicate a larger degree of cold working). As expected, the polished samples can be treated as stress-free. The integral widths of the diffraction lines also indicate that almost no plastic deformation was induced by the polishing process, since the values are similar to the ones of the bulk material, which is unaffected by the surface treatment. Deep-rolled and microblasted samples show maximum RSs of about −800 MPa. While the microblasted sample shows its maximum directly at the surface, deep rolling led to maximum compressive RSs at a depth of about 200 µm. It should be noted that the compressive RSs resulting from deep rolling are directional (i.e., the RSs are significantly higher in the feed direction compared to the rolling direction (Figure 1)). Compressive RSs due to the deep rolling process were introduced up to a depth of approximately 600 µm, whereas for the microblasted sample the range of influence extents into approximately 50 µm depth. Regarding the mean integral width, for the microblasted sample the value dropped from 3.7° to 1.8° within the first 50 µm below the surface, indicating a rather steep cold working gradient. For the deep-rolled sample, the value directly at the surface referred to about 2.4° and decreased continuously with increasing distance to the surface.

**Figure 4:**
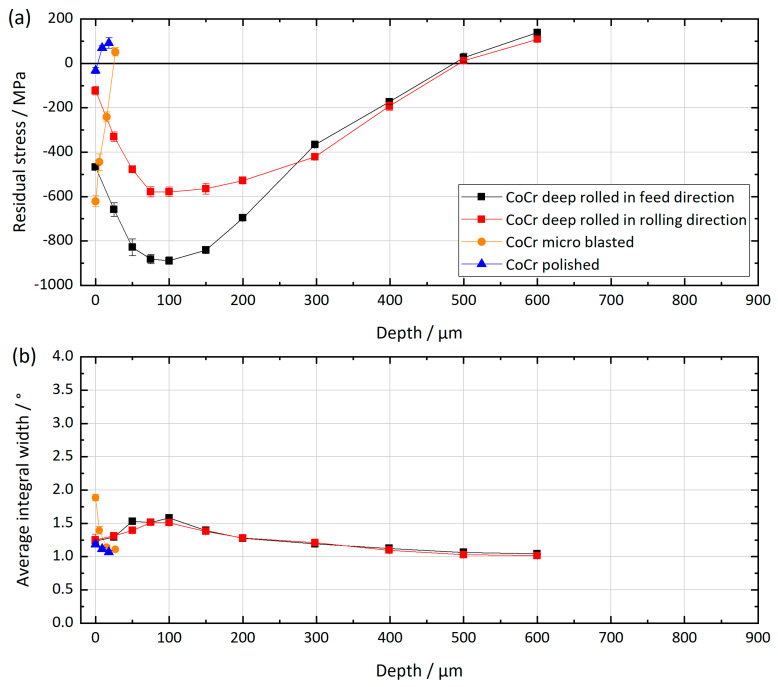
Residual stress depth distributions (**a**) and depths distributions of the average integral widths of the diffraction lines (**b**) for the three differently surface-treated CoCrMo (CoCr) samples.

Figure 4 shows the depth distributions of RSs and mean integral widths for the CoCrMo samples. The courses show similarities to the ones of the Ti6Al4V samples. The polished sample is almost unaffected by the surface treatment, it shows only a small amount of tensile residual stresses close to the surface. Deep rolling and microblasting on the other hand induced characteristic compressive residual stress distributions. Microblasting resulted in compressive RSs of approximately −600 MPa directly at the surface. In addition, a steep gradient with a zero crossing and a change of sign towards tensile RSs in a depth of about 30 µm was found. As for the Ti6Al4V samples, the RSs are higher in the feed direction than in the rolling direction. Directly at the surface, compressive RSs in the feed direction of about −470 MPa and in the rolling direction of about −120 MPa were determined. At a depth of approximately 100 µm, the maximum of the compressive RSs reached about −900 MPa in the feed direction and about –580 MPa in the rolling direction. Zero crossing is observed at a depth of about 500 µm. At depths of more than 500 µm, tensile RSs were determined.

Regarding mean integral widths, the polished sample exhibited values in the range of the unaffected bulk material (see Figure 4). The microblasted sample showed significantly higher values of about 1.9° mean integral width directly at the surface. The value dropped within the first 30 µm below the surface to the values of the unaffected bulk material. The deep-rolled CoCrMo samples exhibited mean integral widths of about 1.2° at the very surface, which is slightly higher than for the unaffected bulk material. The depth profile shows a maximum of about 1.5° in approximately 100 µm depth, which is related to the location of maximal Hertzian stresses. With increasing depth, the integral widths continuously decreased to the value of the unaffected bulk material (1.05°).

### 3.2. Electrochemical Tests 

**Figure 5:**
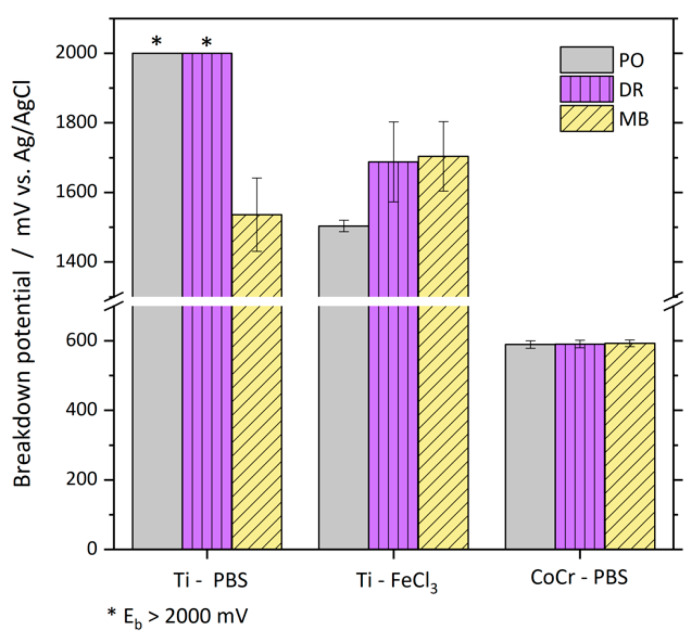
Breakdown potential (E_b_) of polished (PO), deep-rolled (DR) and microblasted (MB) Ti6Al4V samples tested in PBS and in FeCl_3_-containing electrolyte (FeCl_3_) and of polished, deep-rolled and microblasted CoCrMo samples tested in PBS. Values comprise all four anodic polarization scans of three tested samples. Values marked with * refer to breakdown potentials above 2000 mV.

Figure 5 represents the breakdown potentials (E_b_) obtained from anodic polarization curves of the two materials in different surface conditions. For the polished and deep-rolled Ti discs, the breakdown potential could not be reached within the measuring range of up to 2000 mV if the used electrolyte was PBS. The microblasted samples; however, showed a distinct passive layer breakdown potential of 1536 ± 105 mV. If tested in the FeCl_3_-containing electrolyte, the breakdown potential of all three investigated surface conditions was below 2000 mV. Polished samples had the lowest breakdown potential of 1504 ± 17 mV, while E_b_ of deep-rolled and microblasted samples referred to 1688 ± 115 and 1704 ± 100 mV, respectively. The surface treatments did not influence the breakdown potentials of CoCr discs, E_b_ accounted to 589 ± 10, 591 ± 10 and 593 ± 9 mV for polished, deep-rolled and microblasted discs, respectively. The breakdown potentials were hardly influenced by the scan number of the cyclic repeated polarization. 

**Figure 6:**
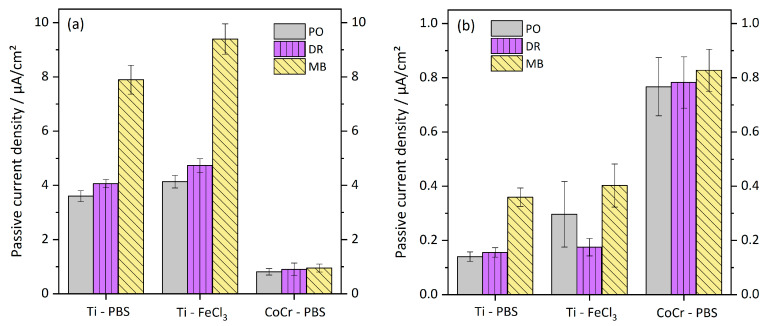
Passive current densities of polished, deep-rolled and microblasted Ti6Al4V samples tested in PBS and in FeCl_3_ and of polished, deep-rolled and microblasted CoCrMo samples tested in PBS. (**a**) Passive currents from the first anodic polarization cycle. (**b**) Passive currents from polarization cycles no. 2–4.

Figure 6 presents the passive current densities from the anodic polarization curves. Passive current densities of the first and the following scans, respectively, are displayed separately because Ti shows clearly increased passive current densities during the first anodic polarization cycle compared to all following scans (cp. Table 2). The difference amounts to about one order of magnitude. Passive current densities of the microblasted Ti samples are about two times higher than those of polished and deep-rolled samples in both tested electrolytes. In PBS, polished and deep-rolled surfaces exhibited comparable passive current densities. If tested in FeCl_3_-containing electrolyte, the polished and deep-rolled samples showed comparable passive current densities in the first polarization cycle, while in the following cycles the deep-rolled surface show a tendency towards lower I_p_. Passive current density of MB Ti samples was raised during the first anodization cycle in the FeCl_3_-containing electrolyte in comparison to PBS. This tendency diminished during repeated polarization. 

Passive current densities of CoCr are less affected by cycle number and surface condition, respectively (cp. Table 2). However, there are slight tendencies towards higher passive current densities during the first anodization and for the microblasted surface, respectively. 

**Figure 7:**
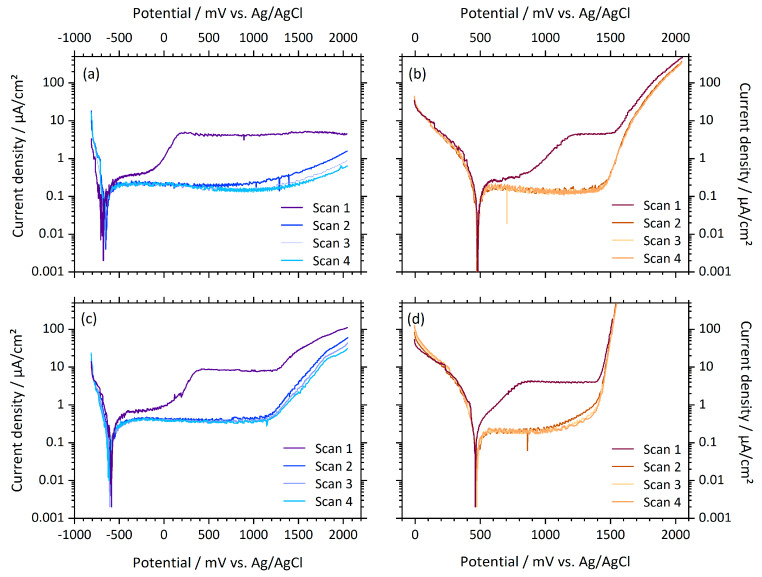
Anodic polarization curves of Ti6Al4V samples. (**a**) Deep-rolled sample tested in PBS, (**b**) deep-rolled sample tested in FeCl_3_, (**c**) microblasted sample tested in PBS and (**d**) polished sample tested in FeCl_3_.

The anodic current density–potential plots shown in Figure 7 illustrate the distinct first polarization cycle of Ti samples. The passive region subdivides in two parts of a less distinct plateau at passive current densities in the range of several hundred nA, and a second, more pronounced plateau in the µA range. In scans no. 2–4, a stable plateau region at the lower passive current densities developed. The FeCl_3_-containing electrolyte resulted further in a displacement of the corrosion potential (i.e., the potential at zero crossing of the current density, which refers to the minimal value in logarithmic representation), from about −600 to about 500 mV (cp. Table 3), which means that the cathodic reaction is enhanced if FeCl_3_-containing electrolyte is used.

**Figure 8:**
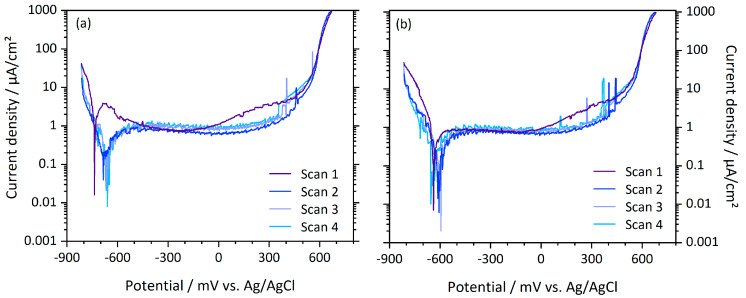
Anodic polarization curves of (**a**) a polished and (**b**) a microblasted CoCrMo sample tested in PBS.

For CoCr discs, again the curve progression in the first polarization cycle differs from the following cycles, as illustrated for a polished and a microblasted CoCr sample, respectively, in Figure 8. For polished and deep-rolled samples, during the first cycle, a passivation peak at approximately −700 mV can be noticed (cp. Figure 8a). After reaching the local maximum, the current density lowers to the passive current densities displayed in Table 2. With further increasing potential starting at approximately 0 mV, the passive current rised again slowly to a shoulder before the breakdown potential—characterized by the abruptly rising current density—is reached. This progression was observed for all mechanical surface treatments.

### 3.3. Surface Roughness

**Figure 9:**
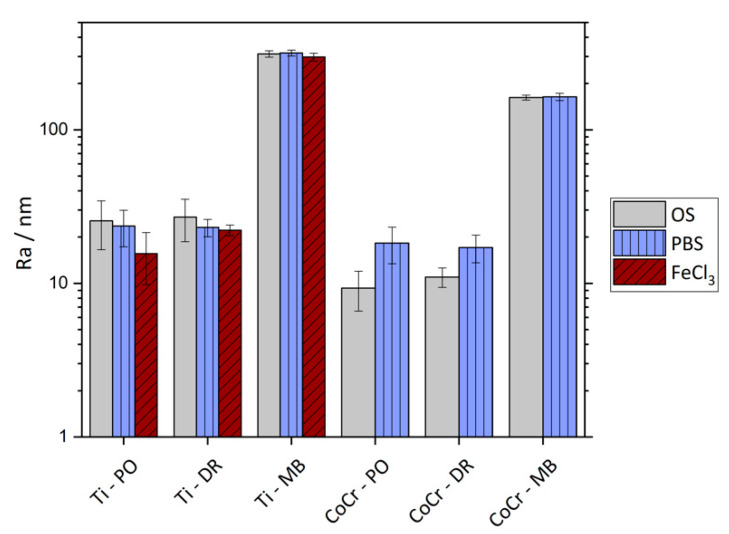
R_a_ values (nm) for Ti6Al4V and CoCrMo in polished, deep-rolled and microblasted surface condition. “Original surface” (OS) refers to the region which had not been in contact with the electrolyte during electrochemical testing; PBS refers to the surface after being electrochemically tested in PBS; FeCl_3_ refers to the surface after being electrochemically tested in FeCl_3_-containing electrolyte.

Figure 9 presents the surface roughness value R_a_ of all tested samples in the as-mechanically- treated condition as well as after electrochemical testing. In Table 4, the R_a_ and R_z_ values are displayed. Generally, Ti samples comprise a higher surface roughness than CoCr samples. The roughness values of polished and deep-rolled samples are comparable, as shown in Table 4. For Ti samples only, deep rolling leads to slightly increased R_z_ values in comparison to polished samples. Microblasting results in a surfaces roughness of about one order of magnitude higher than in case of polishing or deep rolling. These findings are reflected by the SEM images in Figure 2. 

During electrochemical testing, the samples were exposed to the electrolyte in combination with cyclic anodic polarization. Electrochemical testing of Ti samples in PBS did not result in significant changes of the surface roughness of the exposed areas. Polished Ti samples showed; however, a tendency towards decreasing surface roughness values in the regions that have been exposed to the FeCl_3_-containing electrolyte. 

CoCr samples were solely tested in PBS. After electrochemical testing, a slightly increased surface roughness of the polished and deep-rolled surfaces was detected. The microblasted surfaces; however, were not altered by the cyclic anodic polarization, regardless of the sample material or electrolyte. 

## 4. Discussion

Generally, Ti6Al4V showed higher breakdown potentials than CoCrMo, meaning that Ti has a higher resistance to pitting corrosion. At polished and deep-rolled samples, breakdown of the passive layer could not be accomplished in PBS up to a potential of 2000 mV. This is consistent with the literature reporting breakdown potentials between 2500 and 3500 mV for Ti6Al4V in chloride-containing electrolytes [28,29]. The microblasted sample, on the other hand, exhibited a clearly lower breakdown potential of 1536 mV in PBS, which indicates a greater susceptibility to pitting corrosion of microblasted surfaces in comparison to polished or deep-rolled surfaces. Polarization of Ti samples in the FeCl_3_-containing electrolyte led to breakdown of the passive layer for all tested surface conditions well below 2000 mV, while in this corrosion-accelerating electrolyte, the polished samples exhibited passive layer breakdown at lower potential than microblasted and deep-rolled samples. Interestingly, the addition of FeCl_3_ only resulted in decreased breakdown potentials for PO and DR surfaces, the breakdown potential of the microblasted surface was not further lowered by the FeCl_3_-containing environment. 

We consider passive current densities as qualitative measurement for material degradation processes, as these involve exchange and transport of ions and charged molecules. Higher passive current densities would therefore represent increased material degradation rates, while lower passive current densities would refer to smaller corrosion rates. Polished and deep-rolled Ti6Al4V samples showed substantially lower passive current densities than samples with microblasted surfaces, which confirms that the corrosion resistance of Ti6Al4V is higher after metallographical polishing and deep rolling than after microblasting of the surface. 

However, during the first anodic polarization cycle, passive current densities of Ti are substantially higher than passive current densities of CoCr. In addition, we observed two distinct regions in the passive potential range. It is well known that Ti6Al4V spontaneously forms a stable oxide layer when exposed to air. The oxide layer has a thickness of approximately 5 nm and consists mainly of TiO_2_ and a small quantity of TiO and Ti_2_O_3_ at the metal–oxide interface [28]. Milosev et al. showed that upon polarization of Ti6Al4V, the thickness of the TiO_2_ layer increases and Al_2_O_3_ is introduced in the passive layer already at low potentials [28]. We; therefore, attribute the distinctive passive region during the first polarization to growth and chemical transformation of the oxide layer. In all following polarization cycles, the oxide layer stabilized and does not undergo further changes, as can be seen from the constant current density within the passive region and the almost identical current density–potential plots. 

As illustrated in Figure 8, the first polarization cycle of CoCrMo samples differed from all following cycles. For polished and deep-rolled samples, a passivation peak (i.e., an active–passive transition) was observed during the first polarization scan. Hence, we suppose a thickening or healing of the existing oxide layer at low potentials. All surface treatments exhibited increased passive current densities over a potential range of approximately 300 mV just before passive–transpassive transition. This might be attributed to chemical changes, as the composition of the oxide layer is highly dependent on the applied potential [30,31]. The spontaneously formed oxide layer mostly consists of Cr_2_O_3_ and small quantities of Co- and Mo-oxides and—according to Milosev et al.—has a thickness of about 1.8 nm [31]. Chemical modifications caused by polarization in intermediate potential ranges include the incorporation of Co- and Mo-oxides in the passive layer, which is accompanied by an increase in thickness [31]. Milosev et al. found the increase in thickness of the passive layer to account for about a factor 3, if CoCrMo is polarized up to 800 mV (against a saturated calomel electrode) in simulated physiological solution [31]. However, after the first polarization cycle, the passive layer of CoCrMo stabilized, so that, in all following cycles, neither passivation peaks nor increasing passive current densities were observed before passive–transpassive transition. Among the different surface treatments, the passive current densities of CoCrMo hardly varied. The changes within the oxide layer on CoCrMo caused by polarization can; therefore, be considered to be independent of the mechanical surface treatments. 

The decreased corrosion behavior of microblasted Ti6Al4V samples can be attributed to the higher roughness of the surface. A higher corrosion resistance related to lower surface roughness has been reported for Ti6Al4V but also for other metals and alloys, such as cp-Ti and NiTi [29,32,33,34]. The higher passive current densities in microblasted Ti samples can be attributed to the true surface area, which increases with roughness. The irregular grooves that are formed upon microblasting might, in addition, lead to small, enclosed areas with reduced diffusion in the electrolyte. This could lead to a locally-restricted oxygen depletion in the electrolyte, which may result in localized corrosion similar to the process of crevice corrosion [29]. Additionally, the oxide layer of the rough surface may possess a higher degree of imperfections, which would make the surface more susceptible to pitting corrosion [29]. The lower breakdown potential of the microblasted Ti6Al4V samples tested in PBS can be attributed to these effects. In a more aggressive corrosive environment; however, these effects seem to play a minor role, as in the FeCl_3_-containing electrolyte all three Ti6Al4V surface modifications exhibited breakdown potentials in the same range. 

For CoCrMo; however, neither influence of the near-surface RS state nor influence of roughness on corrosion behavior could be observed. The roughness values of microblasted CoCrMo are about two times lower than the roughness values of microblasted Ti. There might be a critical roughness, above which the corrosion behavior is affected. It is possible; however, that the influence of roughness on breakdown potential and passive current densities is alloy dependent. Evidence can be found in literature of metals and alloys that do not show a clear relation of corrosion resistance and surface roughness (e.g., β-Ti alloy and stainless steel) [33,35]. 

In addition to the analysis of the potentiodynamic polarization curves, the roughness of the materials before and after polarization might be an indicator for the extent of the corrosional attack during cyclic polarization. The deep-rolled and polished CoCrMo samples showed a tendency towards increased surface roughness after the four conducted polarization cycles. This indicates material loss due to pitting corrosion. Deep-rolled and microblasted Ti samples did not show altered roughness values after cyclic polarization in PBS and FeCl_3_-containing electrolyte. The polished samples polarized in FeCl_3_-containing electrolyte showed; however, a slight tendency towards decreasing surface roughness after the electrochemical tests. Roughness decrease due to electrochemical material degradation can be explained by the difference in Volta potential between valley and peaks, as electrons in peak areas are more likely to escape the material than electrons in valley areas [36]. This would mean that roughness progression due to galvanic corrosion would pass a minimum before a roughness increase due to pitting becomes evident. Furthermore, in the FeCl_3_-containing environment, polished Ti samples exhibited lower breakdown potential than deep-rolled Ti samples. This indicates that the corrosion resistance of deep-rolled Ti6Al4V might be superior to that of polished Ti6Al4V, which might be correlated to the high compressive RSs in the near-surface region. Thus, for the three investigated surface conditions of Ti6Al4V, deep rolling resulted in relatively higher corrosion resistance. 

This work investigated the potential of surface treatments for enhancing the corrosion resistance of passive metals, which are applied in the field of surgical implants. It has been shown that the surface treatments did impact the corrosion behavior of the metals, even though the observed differences on the breakdown potentials and passive current densities between polished and deep-rolled samples were rather small. The corrosion of taper connections is; however, mainly initiated by occurring micromotion, which can damage the passive layer leading, in turn, to fretting and galvanic corrosion [3,37,38]. The mechanical resistance of the oxide layer is; therefore, crucial for the corrosion resistance of the applied alloys. Assessment of corrosion in this study was somehow simplified, as the mechanical component of taper corrosion was not taken into account. This was done in order to separate the effects of occurring micromotion from the effects caused by galvanic corrosion. In this setup, the corrosion behavior is; therefore, rather governed by sample roughness than by the induced compressive RSs, especially since the investigated alloys are passive metals with a naturally high corrosion resistance. However, apart from the slightly improved corrosion resistance for the deep-rolled surface of Ti6Al4V observed in this study, the induction of compressive RSs by means of deep rolling have moreover the potential of combining a mirror smooth sample surface with the beneficial effects in terms of fretting and fatigue resistance. 

Further studies are needed that involve actually occurring forces and resulting micromotion in order to evaluate the impact of compressive RSs onto the corrosion resistance of modular taper connections.

## 5. Conclusions 

Compressive RSs were introduced in Ti6Al4V and CoCrMo by means of deep rolling and microblasting, while metallographically-prepared samples served as reference. For CoCrMo, the surface treatments did not alter the corrosion behavior. For Ti6Al4V, microblasting led to a lowered breakdown potential and/or increased passive current densities, which indicates decreased corrosion resistance. Further studies comprising mechanical loads are needed in order to assess the impact of mechanical surface treatments with respect to corrosion of modular taper connections in total joint arthroplasty. 

## Figures and Tables

**Table 1:** Processing parameters applied for deep rolling and microblasting.

	Parameters Deep Rolling	Parameters Microblasting
Ball material / diameter	Ceramic / 6 mm	Glass beads MS 550B / 20–30 µm
Pressure	180 bar	3 bar
Feed rate	2000 mm/min	8 mm/s
Line spacing between traces	0.04 mm	1 mm
Initial distance to surface	–	10 mm
Coverage ratio	–	100%
Flow rate	–	1.5 kg/min

**Table 2:** Passive current densities (mean (SD)) during first anodic polarization cycles and anodic polarization cycles no. 2–4 of polished, deep-rolled and microblasted Ti6Al4V and CoCrMo samples.

	Cycle 1 / nAcm^−2^			Cycles no. 2–4 / nAcm^−2^		
	PO	DR	MB	PO	DR	MB
Ti-PBS	3600 (200)	4067 (153)	7900 (529)	140 (17)	156 (17)	359 (34)
Ti-FeCl_3_	4133 (231)	4733 (252)	9400 (557)	297 (11)	175 (6)	403 (79)
CoCr-PBS	813 (118)	908 (225)	950 (145)	767 (107)	783 (95)	828 (78)

**Table 3:** Corrosion potential (mean (SD)) of polished, deep-rolled and microblasted Ti6Al4V specimens tested in PBS and FeCl_3_-containing electrolyte, respectively.

	PO / mV	DR / mV	MB / mV
PBS	−615 (94)	−642 (71)	−566 (30)
FeCl_3_	498 (27)	469 (25)	461 (31)

**Table 4:** R_a_ and R_z_ values in nm for all tested materials, surface conditions and electrolytes.

	Ti6Al4V / nm			CoCrMo / nm		
	PO	DR	MB	PO	DR	MB
R_a_ / OS	25.5 (8.9)	27.0 (8.3)	311.9 (15.1)	9.3 (2.7)	11.0 (1.6)	162.2 (6.0)
R_a_ / PBS	23.6 (6.3)	23.1 (3.0)	315.9 (14.2)	18.3 (4.9)	17.1 (3.5)	163.7 (8.9)
R_a_ / FeCl_3_	15.6 (5.8)	22.2 (1.8)	297.5 (17.6)	–	–	–
R_z_ / OS	177.9 (63.3)	210.1 (53.4)	2004 (131)	68.5 (20.4)	69.1 (17.5)	1077 (91)
R_z_ / PBS	178.3 (61.6)	200.5 (28.7)	2010 (160)	122.7 (23.9)	106.4 (15.4)	1097 (89)
R_z_ / FeCl_3_	109.5 (32.4)	192.8 (22.5)	1883 (115)	–	–	–
